# Factors influencing higher vocational nursing students’ mental health during internships: a cross-sectional study

**DOI:** 10.3389/fpubh.2025.1700661

**Published:** 2025-11-14

**Authors:** Dan Wang, Yalin Mao, Xiangguang He, Tian Li, Huping Gong

**Affiliations:** 1 Intelligent Healthcare Services and Management for the Elderly, Binzhou Polytechnic, Binzhou, China; 2 Jiangsu College of Safety Technology, Xuzhou, Jiangsu, China; 3 School of Nursing, The Philippine Women’s University, Manila, Philippines; 4 College of Rehabilitation, Gannan Medical University, Ganzhou, China; 5 College of Nursing, Gannan Medical University, Ganzhou, China

**Keywords:** nursing students, clinical internship, mental health, influencing factors, psychological distress

## Abstract

**Background:**

Internship is an important transition stage for nursing students from school to clinical work. However, students often suffer from mental health problems such as depression, anxiety and stress during internship, which further affects the effect of internship and career choice.

**Aim:**

This study aims to explore the mental health problems of higher vocational nursing students and its influencing factors.

**Methods:**

This study used a cross-sectional design to investigate 432 nursing students during internship who were studying at Binzhou Polytechnic from May to June 2025. The general information questionnaire, General Self-Efficacy Scale (GSES) and Depression, Anxiety and Stress Scale (DASS-21) were used to collect data to understand the mental health status of higher vocational nursing interns and analyze the influence of different factors on mental health problems.

**Results:**

24.7% of the nursing interns had depression problems, 26.5% had anxiety problems, and 22.8% felt great pressure. Binary logistic regression analysis showed that internship in a tertiary grade A hospital and male students were associated with an increased risk of psychological problems (OR > 1). However, often or sometimes feeling cared for during internship, liking for the nursing profession, thinking that the nursing profession was respected, and high self-efficacy were associated with reduced risk of psychological problems (OR < 1). Conclusions: Hospital, gender, liking for the nursing profession, thinking that the nursing profession was respected, feeling cared for during internship and self-efficacy were all significantly associated with the mental health of higher vocational nursing students during internship. These findings provide a basis for the development of targeted mental health interventions to help improve the mental health of nursing students.

## Introduction

Internships serve as a critical transitional stage for nursing students as they move from academic learning to clinical practice. They only play an important role in professional development, but also act as a bridge connecting theoretical knowledge with practical skills (1). Internships are thus essential for nursing students, and their process and effectiveness have a profound impact on their future clinical performance and career adaptability (2). During internships, nursing students not only face unfamiliar and complex clinical environments, but also have to deal with several challenges, such as effectively applying the knowledge and skills learned in the classroom to real clinical situations, and dealing with complex interpersonal interactions with patients, patients’ families, and medical team members (3–5). These challenges may induce anxiety, tension, and irritability, which may affect their internship experience and mental health. Some nursing students may experience significant occupational pressure after clinical practice, and when their professional ideals do not align with reality, they may choose to leave the nursing major, leading to a considerable loss of nursing talent (6).

According to the World Health Organization, mental health is an important component of health (7). Mental health refers not only to the absence of mental illness, but also to the ability of individuals to realize their potential, cope with stressors in their lives, work effectively and contribute to society (8). In China, the imbalance between the rapidly advancing medical system and the huge demand for health care services has resulted in medical staff being in a state of heavy workload for a prolonged period (9). In addition, the country is facing significant challenges such as nursing staff shortage and a high turnover rate (10). As future healthcare professionals, the mental health of nursing students not only affects their personal development but is also closely linked to patient safety and the stability of the nursing workforce. Research indicates that nursing students under significant psychological stress are more prone to academic burnout and face a notably higher risk of clinical errors (11). In recent years, the phenomenon of mental health problems among higher vocational nursing students during internships has attracted significant attention from researchers. These students experience stress, anxiety, and depression, and the incidence of these issues is increasing (11, 12).

Unfortunately, a considerable number of nursing students fail to actively seek appropriate psychological assistance. Previous studies have identified a variety of factors that affect the mental health of nursing interns, including the complexity of the internship environment, work intensity, lack of proficiency in professional skills, low self-efficacy, and uncertainty about future career prospects (3, 4, 13). Specifically, “lack of proficiency in professional skills” refers to clinical inexperience and inadequate emergency judgment, reflecting difficulties in applying theoretical knowledge to practice (14). “Low self-efficacy” diminishes their confidence when facing professional pressures, thereby weakening their psychological capacity to cope with challenges (15). Furthermore, “work intensity” encompasses not only long hours and high task density but also the emotional exhaustion from caregiving, which can lead to prolonged physical and mental fatigue (16). These factors, intertwined with a complex training environment and career uncertainties, collectively pose substantial challenges to the mental well-being of nursing interns.

Although several studies have explored mental health problems in nursing interns, most of them have focused on a single mental health indicator such as depression, anxiety, or stress, and therefore, there is a lack of research on the coexistence of multiple mental health problems, and more importantly, on their underlying determinants. Thus, in this study, we aimed to explore the determinants influencing anxiety, depression, and stress among nursing students during internships, and provide valuable insights for nursing educators and medical institutions to improve nursing students’ internship experience and mental health outcomes.

## Method

### Study design and participants

This cross-sectional study was conducted at a higher vocational college in China (Binzhou, Shandong province, China) between May 16 and June 30, 2025. In China, higher vocational colleges play a key role in training frontline clinical nurses, and the mental health status of their interns is crucial to the stability of the nursing workforce. Approximately 500–700 students from the Department of Nursing at this higher vocational college enter the clinical practice stage each year. The internship hospitals mainly include general hospitals at the secondary level or above in Shandong Province, and all the hospitals are certified as clinical teaching bases by the education department. Thus, our study setting effectively represents the typical clinical learning environment and psychological stress faced by vocational nursing intern groups under the local allocation of medical resources. All higher vocational nursing students participating in clinical internships received a questionnaire using Wenjuanxing (https://www.wjx.cn/, a widely used online survey platform in China), a widely used online survey platform in China.

The inclusion criteria for this study were as follows: (1) full-time vocational nursing students at the stage of clinical internship; (2) having more than 1 month of clinical internship experience; and (3) the willing to participate in the study and provide informed consent. Exclusion criteria included nursing students who took sick leave or personal leave for more than 1 month during the clinical internship.

**Figure 1:**
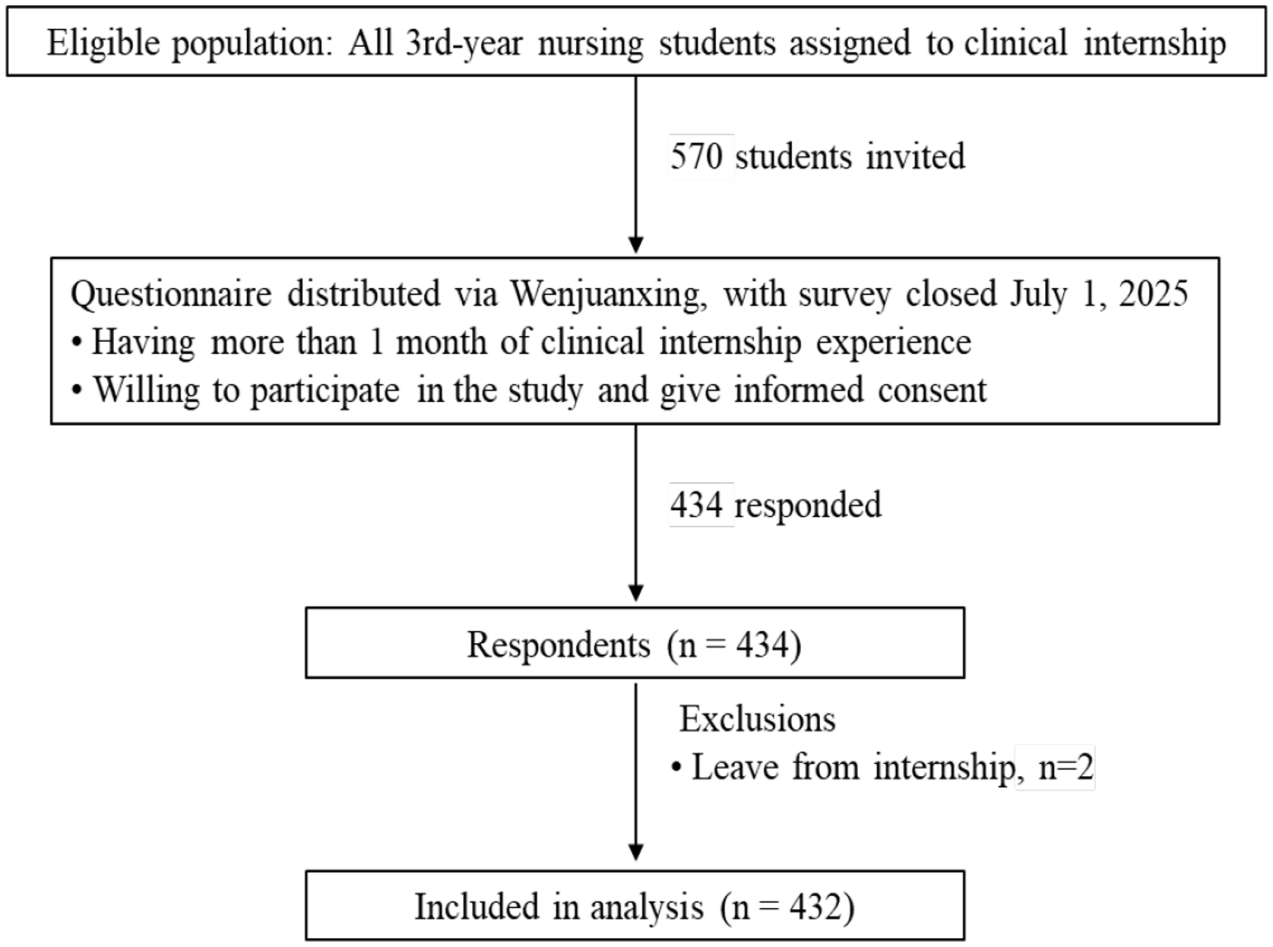
Flowchart of study participants.

Nursing students could only submit the questionnaire once after responding to all questions. The flowchart is shown in Figure 1.

### Ethical considerations

This study was approved by the Ethics Review Committee of Binzhou Polytechnic (2025001). All participants provided written informed consent prior to participation. All data were strictly anonymized to protect privacy of the participants. The data were used only in this study.

### Measures

The study questionnaire consisted of three parts: a self-designed questionnaire, the General Self-Efficacy Scale (GSES), and the Depression, Anxiety, and Stress Scale (DASS-21).

The self-designed questionnaire adopted in this study was used to understand the basic information of students and their views related to the nursing profession, and to analyze whether these factors had an impact on students’ mental health problems. It specifically included 10 questions on age, gender, only-child status, place of origin, class cadre, grade of the internship hospital, feeling cared for during the internship, liking the nursing profession, perception of professional respect, and receiving assistance from one’s family.

The GSES adopted in this study was used to evaluate the self-efficacy level of the nursing students. This scale was developed by Schwarzer (17) and consists of 10 items. It uses a 4-point Likert scale (1–4 points), with the total score being the average of the 10 items, ranging from 1 to 4 points. The higher the score, the stronger the overall confidence of the individual in dealing with challenges. The Chinese version of this scale has good reliability and validity, serving as a reliable tool for educational intervention and mental health research (18). In this study, the Cronbach’s alpha coefficient of the GSES was 0.989. Item analysis confirmed uniformly high corrected item-total correlations for all items (range: 0.905–0.966), and the alpha value did not increase meaningfully with the deletion of any item. The details are shown in Supplementary Table S1.

In this study, the DASS-21 was used to evaluate the mental health status of nursing interns in higher vocational colleges. This scale was developed by Lovibond PF and Lovibond SH (19), and it is a simplified version of the original DASS-42 scale (20), assessing emotional experiences within the past 2 weeks. It contains 21 items and is divided into three sub-scales: Depression (DASS-21-D), anxiety (DASS-21-A), and stress (DASS-21-S). Each dimension contains seven items, and is scored using a 4-point Likert scale. The score range is from 0 (“not applicable to me at all “) to 3 (“very applicable to me “). According to the usage guidelines of DASS-21, in order to ensure that the scoring results correspond to the original DASS-42, the total scores of each sub-scale need to be multiplied by 2. The total score range of each dimension is 0–42. The higher the score, the higher the corresponding emotional distress level of the individual (21). The Chinese version of DASS-21, which has undergone cross-cultural translation and localization, has demonstrated good reliability among Chinese students (22, 23). A depression score ≤9 was defined as normal (no depression), and >9 was defined as depression (10–13 as mild depression, 14–20 as moderate depression, 21–27 as severe depression, and ≥28 as very severe depression). Anxiety score ≤7 was defined as normal (no anxiety), and >7 was defined as anxiety (8–9 as mild anxiety, 10–14 as moderate anxiety, 15–19 as severe anxiety, and ≥20 as very severe anxiety). Stress scores ≤14 were classified as normal, and >14 were defined as stress (15–18 as mild stress, 19–25 as moderate stress, 26–33 as severe stress, and ≥34 as very severe stress) (24–26). Therefore, the mental health problems were categorized as “no” (having no depression, no anxiety, and no stress) and “yes” (having depression, anxiety, or stress) (26). In this study, the Cronbach’s alpha coefficients for the depression, anxiety, and stress were 0.982, 0.980, and 0.977, respectively. Item analysis confirmed uniformly high corrected item-total correlations for all items (range: 0.884–0.963), and the alpha value did not increase significantly with the deletion of any item. The details are presented in Supplementary Table S2.

### Determination of sample size

The sample size for this study was calculated *a priori* to ensure the robustness of the planned binary logistic regression analysis with 11 independent variables. We employed the established “events per variable” criterion for logistic regression, which recommends 10–15 events per predictor to ensure reliable parameter estimates (27, 28). To adopt a conservative criterion of 15 events per variable, a minimum of 165 events (i.e., participants with a mental health problem) was required. The outcome was a composite variable indicating the presence of at least one DASS-21-defined condition (depression, anxiety, or stress). To estimate the expected event rate, we synthesized prevalence rates reported in prior literature, which showed rates of approximately 41.7% for anxiety, 28.7% for depression, and 20.2% for stress in comparable populations (29). Assuming no comorbidity for a conservative calculation, the composite outcome prevalence was projected to be 66.8%. The minimum total sample size was therefore calculated as 165 / 0.668 ≈ 247. To accommodate potential incomplete responses, we targeted a final sample of 270 participants. Ultimately, the study successfully included 432 participants in the final analysis, a sample size that substantially exceeds the calculated minimum requirement.

### Data analysis

The mental health status of vocational college nursing students was described using percentages, and the self-efficacy score was described as the mean ± the standard deviation. The chi-square (χ^2^) test was used to analyze differences in mental health problems among nursing students with different characteristics, and the Spearman’s correlation analysis was used to analyze the correlation between self-efficacy and mental health problems. Finally, the influence of different characteristics on mental health problems was analyzed through binary logistic regression, and the results were presented in a forest plot. The internal consistency reliability of the scales used in this study (GSES and DASS-21) was assessed by calculating Cronbach’s alpha coefficients. Furthermore, to thoroughly investigate the psychometric properties, corrected item-total correlations and Cronbach’s alpha-if-item-deleted values were computed for all scale items to examine each item’s contribution to the total score and to check for potential item redundancy. All statistical analyses were performed using IBM SPSS Statistics Version 26, and the forest plot was performed using R software Version 4.4.2.

## Result

### Status of mental health problems

The mental health problems of students mainly included depression, anxiety and stress. The number of people experiencing depression was 107 (24.7%). The number of people experiencing anxiety was 115 (26.5%). The number of people experiencing stress was 99 (22.8%). The detail information was shown in Table 1.

**Table 1:** Status of mental health problems of vocational college nursing students, *n* (%).

Mental health problem	Normal	Mild	Moderate	Severe	Very severe
Depression	327 (75.3%)	22 (5.1%)	43 (9.9%)	4 (0.9%)	38 (8.8%)
Anxiety	319 (73.5%)	3 (0.7%)	55 (12.7%)	13 (3.0%)	44 (10.1%)
Stress	335 (77.2%)	10 (2.3%)	9 (2.1%)	11 (2.5%)	30 (6.9%)

### Association between different characteristics and mental health problems

The χ^2^ test showed in Table 2 that the grade of the internship hospital, gender, feeling cared for during the internship, liking the nursing profession, perception of professional respect, and receiving assistance from one’s family were related to the mental health problems of vocational college nursing interns (*p* < 0.05), while age, only-child status, place of origin, and class cadre were not related (*p* > 0.05).

**Table 2:** Association between different characteristics and mental health problems in higher vocational college nursing students.

Group	Mental health problems		*X* ^2^	*p*
	No	Yes		
Grade of the internship hospital			6.422	0.040
Tertiary Grade A hospital	208	91		
Tertiary Grade B hospital	26	12		
Secondly Grade A hospital	80	17		
Gender			6.479	0.011
Male	79	45		
Female	235	75		
Age, years old			0.001	0.973
18–21	277	106		
22–28	37	14		
Only-child status			2.369	0.124
Yes	60	31		
No	254	89		
Class cadre			2.142	0.143
Yes	87	25		
No	227	95		
Liking the nursing profession			25.940	<0.001
Like	163	35		
General	136	66		
Dislike	15			
Place of origin			0.153	0.696
City	78	32		
Country	236	88		
Perception of professional respect			30.642	<0.001
Respected	181	45		
General	122	54		
Not respected	11	21		
Receiving assistance from one’s family			6.306	0.043
Full help	196	61		
A little help	95	43		
Helpless	23	16		
Feeling cared for during internship			37.943	<0.001
Often cared about	208	53		
Sometimes cared about	100	48		
Lack of cared about	6	19		

### Association between self-efficacy and mental health problems

The average score of the self-efficacy was 3.08 ± 0.93, at a moderately high level. The results of Spearman correlation analysis showed in Table 3 that there was a negative correlation between the self-efficacy and mental health problems in vocational college nursing students.

**Table 3:** Correlation between self-efficacy and mental health problems in nursing students(r).

Category	Self-efficacy	*p*
Depression	−0.201	<0.001
Anxiety	−0.207	<0.001
Stress	−0.203	<0.001
Mental health problems	−0.198	<0.001

### Influencing factors of mental health problems in higher vocational college nursing students

After conducting a binary logistic regression analysis on the mental health problems of vocational nursing students (Table 4), it was found that the grade of the internship hospital, gender, liking the nursing profession, feeling cared for during internship, perception of professional respect, and self-efficacy were significantly correlated with the mental health problems of higher vocational college nursing students during internship.

**Table 4:** Binary logistic regression analysis for mental health problems in nursing students.

Variable	β	Standard error	Wald *χ*^2^	OR (95% CI)	*p*-value
Grade of the internship hospital					
Second grade A hospital (Ref)				1.0 (reference)	
Tertiary grade A hospital	0.802	0.339	5.578	2.23 (1.15–4.34)	0.018*
Tertiary grade B hospital	0.943	0.492	3.673	2.57 (0.98–6.74)	0.055
Gender					
Female (Ref)				1.0 (reference)	
Male	0.762	0.262	8.457	2.14 (1.28–3.58)	0.004*
Liking the nursing profession					
Dislike (Ref)				1.0 (reference)	
Like	−1.013	0.488	4.307	0.36 (0.14–0.94)	0.038*
General	−0.381	0.451	0.714	0.68 (0.28–1.65)	0.398
Feeling cared for during internship					
Lack of care about (Ref)				1.0 (reference)	
Often cared about	−1.942	0.597	10.579	0.14 (0.04–0.46)	0.001*
Sometimes cared about	−1.639	0.583	7.907	0.19 (0.06–0.61)	0.005*
Perception of professional respect					
Not respected (Ref)				1.0 (reference)	
Respected	−1.195	0.518	5.316	0.30 (0.11–0.84)	0.021*
General	−0.945	0.472	4.002	0.39 (0.15–0.98)	0.045*
Receiving assistance from one’s family					
Helpless (Ref)				1.0 (reference)	
Full help	0.377	0.471	0.642	1.46 (0.58–3.67)	0.423
A little help	0.120	0.460	0.068	1.13 (0.46–2.77)	0.794
Self-efficacy (Continuous)	−0.259	0.128	4.072	0.77 (0.60–0.99)	0.044*

*p-value < 0.05.

**Figure 2:**
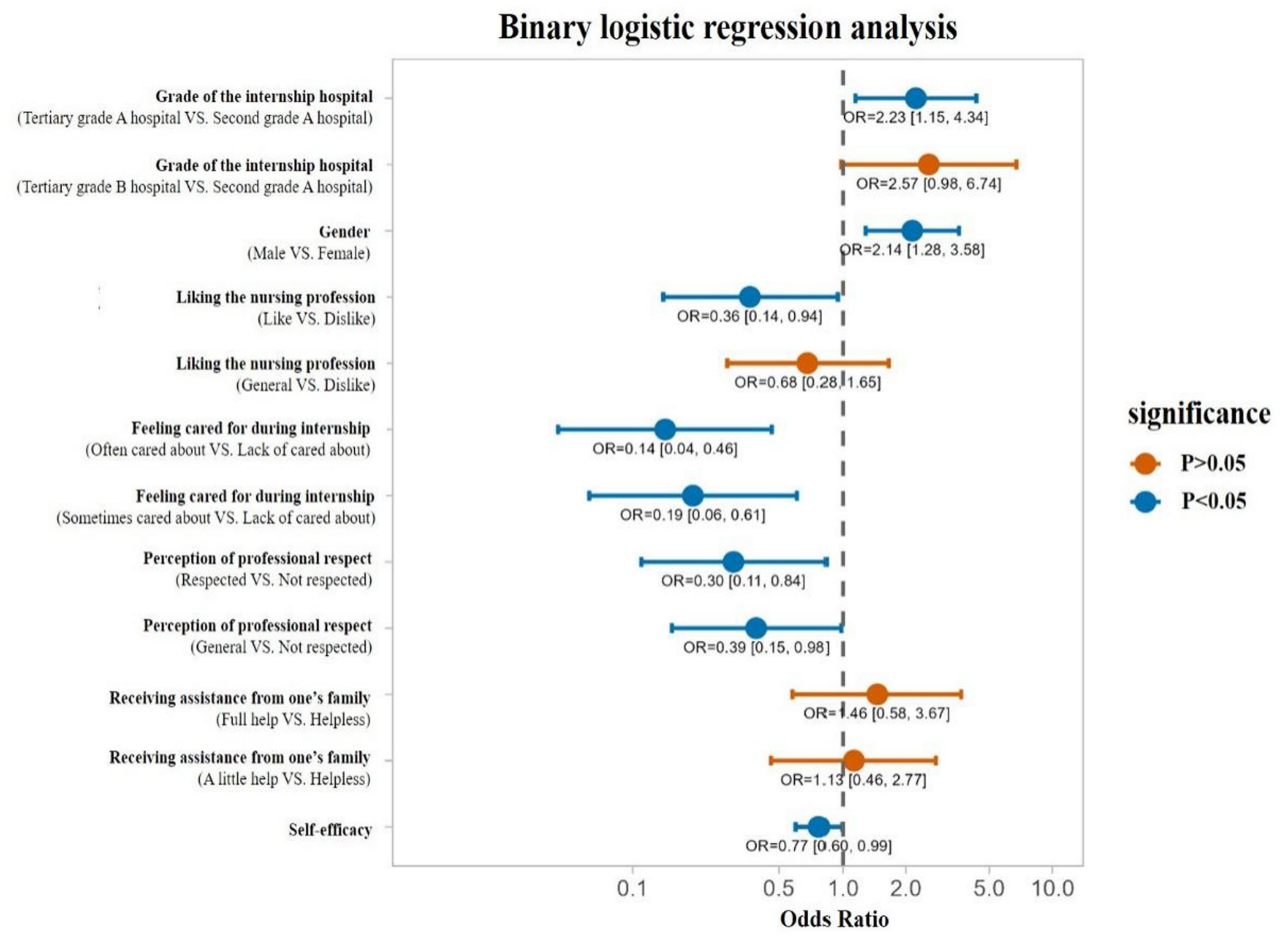
Binary logistic regression analysis of mental health problems in higher vocational college nursing students.

Internships in tertiary grade A hospitals, and being male were associated with an increased risk of mental health problems (odds ratio, OR >1); While liking the nursing profession, often or sometimes feeling cared for during internships, perception of nursing profession was respected, and having a high self-efficacy were associated with a reduced risk of psychological problems (OR <1). The detail was presented with a forest plot in Figure 2.

## Discussion

Our study found that depression (24.7%), anxiety (26.5%), and stress (22.8%) were the predominant mental health problems among higher vocational nursing students during the internship. Clinical practice in a tertiary grade A hospital, male, dislike of the nursing profession, feeling a lack of care during internship, and perceiving that the nursing profession is not respected were factors associated with an increased risk of psychological problems. These factors included elements related to the clinical environment, individual characteristics, and social support.

### Clinical environment factors

Nursing students experience varying degrees of mental health problems during an internship, among which very severe depression and anxiety account for a relatively high proportion. This may be closely related to the high pressure of nursing work, the difficulty of adapting to the internship environment, and the complex interpersonal relationships (3, 30). Nursing work has a certain degree of risk and uncertainty. When interns face changes in patients’ conditions, complex nursing operations, and various rules and regulations of the hospital, they are prone to significant psychological pressure. If the anxiety they experience is not promptly alleviated and supported, it may further worsen and develop into more serious mental health problems (31, 32). Therefore, schools and hospitals should pay attention to the mental health of interns. This can be done by conducting mental health literacy training at the beginning of the internship; establishing confidential and accessible consultation channels; and finally, creating a supportive peer guidance network and a mental health support system to help interns relieve their psychological pressure and improve their mental health, enabling them to better adapt to nursing practice work. These specific steps are crucial for cultivating interns’ psychological abilities to cope with clinical challenges and promoting their long-term professional development.

The incidence of mental health problems among interns in tertiary hospitals is relatively higher than that in secondary hospitals. This may be due to the high work intensity of tertiary hospitals and higher requirements for interns, who need to face more complex clinical situations and higher work pressure (33). Tertiary hospitals require higher levels of professional skills and knowledge, and interns need to quickly adapt to complex clinical environments and deal with multiple emergencies in a short period of time, which may lead to anxiety and stress during their internship. At the same time, clinical instructors in tertiary hospitals usually have high expectations for interns, which may further aggravate the psychological burden of interns (34). A positive, supportive work environment can enhance the self-confidence and sense of belonging of interns (35). To implement this concept, hospital management departments can refer to effective practical experience from both domestic and foreign sources. Existing research has shown that implementing a systematic “clinical practice transition plan” can effectively help interns alleviate the stress of adapting to clinical practice (31). In addition, implementing a standardized clinical mentorship system with experienced mentors, who will not only provide guidance on professional skills, but also pay attention to the psychological adjustment and social integration of interns, will provide them with comprehensive support (36). By implementing a series of measures to optimize work arrangements and improve internship environments, hospitals can better safeguard the physical and mental health of interns.

### Individual factors

In nursing work, physical strength, such as patient handling and prolonged standing, poses challenges for all nurses, but data show that the incidence of mental health problems among male nursing interns is significantly higher than that of their female peers. This phenomenon may be related to the special social and psychological pressures faced by men, particularly when physical exhaustion conflicts with society’s expectations of male gender roles as “strong and resilient” (37). Meanwhile, males may be reluctant to seek help for mental health or emotional problems as this may be seen as a sign of vulnerability (38). Therefore, gender is an important factor affecting the mental health of nursing interns. In mental health activities, psychological support should be provided without associating stress with “vulnerability,” and tasks should be allocated reasonably to alleviate male interns’ concerns about seeking help.

Compared with nursing students who dislike the nursing profession, nursing students who like the nursing profession had the lowest incidence of mental health problems, which indicates that the degree of affinity for the nursing profession can affect the psychological state of nursing students in the process of internship (39). Liking the nursing profession can make students more motivated to overcome difficulties in practice and maintain good mental health (39). Educational institutions and hospitals also play important roles in professional learning. High-quality nursing education can stimulate students’ interest and enthusiasm for the profession and help them better adapt to the internship environment (40). Students who think that the nursing profession is respected had a lower incidence of mental health problems (41). When students think that the nursing profession is respected, their sense of self-belonging is enhanced, which is conducive to mental health. Conversely, if students feel that their profession is not respected, these negative emotions can lead to increased stress, anxiety, and depression, which are extremely detrimental to mental health (42).

Our findings revealed that self-efficacy plays an important protective role in the mental health of nursing interns^,^ aligning with existing literature (43). Nursing students with high self-efficacy are more likely to adopt positive coping strategies and maintain good mental health when facing difficulties and challenges during their internship (44, 45). Students with a high level of self-efficacy can more likely to reframe clinical mistakes or failures as opportunities for growth. This adaptive coping strategy helps mitigate associated psychological responses, such as anxiety, shame and helplessness, by identifying growth opportunities from mistakes or failures in clinical practice (44). At the same time, when facing work pressure, interpersonal conflict, and role adaptation challenges, interns with high self-efficacy are more likely to use positive coping styles, such as taking the initiative to solve problems, seeking support and self-adjusting, thereby effectively reducing their psychological burden (45).

### Social support factors

Nursing interns who often felt often cared for during the internship reported the lowest levels of mental health problems. This highlights the importance of care from others (such as classmates, teachers, colleagues, etc.) on the mental health of interns (30). Such care provides a sense of warmth and support and relieves psychological pressure. The encouragement and guidance of teachers can help interns better cope with complex clinical situations, while the mutual support between classmates can reduce their loneliness and pressure (46). Nursing students who are cared for are more likely to build good networks of relationships that not only provide support during internships, but also lay a foundation for their future career development (47). A good interpersonal network can enhance the sense of social support of interns and reduce job burnout and psychological stress (48).

Although the GSES and DASS-21 are internationally recognized mature scales, the ultra-high reliability coefficients (>0.98) observed in this study strongly suggest that there may be factors contributing to the high consistency of response patterns in the specific sample of this study. This may be related to the sample traits of this study. The students were drawn from the same class in the same school, a group that is highly similar in age, educational background, and current academic stress, possibly causing them to show extremely low response variation when answering psychological scales, thus artificially inflating Cronbach’s alpha. Future studies could validate this phenomenon in more diverse samples.

### Limitation

Although this study revealed the current status and possible influencing factors of mental health problems among vocational nursing students during their internship, solving the mental health problems of nursing students requires joint efforts from multiple systems. But there are also several limitations. First, the cross-sectional design can only reflect the situation at a certain point in time and cannot track the dynamic changes in psychological states, nor can it make causal inferences about influencing factors and outcomes. Second, the sample size of this study was limited, and it mainly focused on nursing students in a specific region, which requires caution when generalizing research conclusions to different background colleges and regions. In addition, data were collected entirely through student self-assessment, which is susceptible to interference from subjective memory and social expectations; the survey was conducted using the “Wenjuanxing” online platform, which is convenient but also has potential issues, such as inconsistent response environments and difficulty in verifying authenticity. Future research should expand the sample size to verify in different regions and cultural backgrounds, and adopt a cohort study design to explore the impact of mental health problems on career selection and work of higher vocational nursing students during internship.

### Implications for nursing education

This study provides valuable insights for nursing educators and medical institutions, highlighting the importance of establishing a well-developed mental health support system. Providing psychological counseling services and enhancing interns’ interest in and commitment to the nursing profession can help alleviate their psychological stress and improve mental health. At the same time, improving the self-efficacy of interns is also an important strategy to enhance their mental health.

## Conclusion

In this study, we found that the higher vocational nursing students mainly experienced depression, anxiety, and stress during internship. Hospital level, gender, liking the nursing profession, perception of professional respect, sense of being cared for during the internship, and self-efficacy were factors significantly associated with mental health problems. The interactions among these influencing factors requires a joint response from multiple systems in order to solve the mental health problems of these nursing students. In this collaborative framework, schools and hospitals need to clarify the division of responsibilities: schools should focus on strengthening students’ psychological preparation and clinical coping skills training before internships, while the primary responsibility for creating a supportive internship environment lies with medical and health institutions. We urge hospitals to implement structural intervention measures, reasonably allocate workloads, establish clinical exclusive mentors, and provide other supportive programs to effectively alleviate work-related stressors during internships. By adopting a model of shared responsibility, the incidence of mental health problems can be effectively reduced, ensuring the smooth completion of clinical internships for vocational nursing students.

## Data Availability

The original contributions presented in the study are included in the article/Supplementary material, further inquiries can be directed to the corresponding author.
