# Supplementary material for: Factors influencing higher vocational nursing students’ mental health during internships: a cross-sectional study
Source: Front Public Health. 2025 Nov 14;13:1700661. doi: 10.3389/fpubh.2025.1700661 (PMC12660294; doi:10.3389/fpubh.2025.1700661)
Supplement: Supplementary file 1 [file Table_1.docx]

| **Table S1 Item-Total Statistics of the General Self-Efficacy Scale (GSES)** | | | | |
| --- | --- | --- | --- | --- |
| Items | Scale Mean if Item Deleted | Scale Variance if Item Deleted | Corrected Item-Total Correlation | Cronbach's Alpha if Item Deleted |
| Self-efficacy-Q1 | 27.59 | 70.251 | .928 | .988 |
| Self-efficacy-Q2 | 27.69 | 69.957 | .905 | .989 |
| Self-efficacy-Q3 | 27.76 | 69.432 | .907 | .989 |
| Self-efficacy-Q4 | 27.71 | 69.145 | .952 | .987 |
| Self-efficacy-Q5 | 27.77 | 69.685 | .939 | .988 |
| Self-efficacy-Q6 | 27.66 | 69.823 | .966 | .987 |
| Self-efficacy-Q7 | 27.66 | 69.944 | .954 | .987 |
| Self-efficacy-Q8 | 27.68 | 70.070 | .950 | .987 |
| Self-efficacy-Q9 | 27.66 | 69.970 | .965 | .987 |
| Self-efficacy-Q10 | 27.71 | 69.750 | .962 | .987 |

| **Table S2 Item-Total Statistics of the Depression Anxiety Stress Scales-21 (DASS-21)** | | | | |
| --- | --- | --- | --- | --- |
| Items | Scale Mean if Item Deleted | Scale Variance if Item Deleted | Corrected Item-Total Correlation | Cronbach's Alpha if Item Deleted |
| DASS-21 Q1 | 29.20 | 274.927 | .902 | .993 |
| DASS-21 Q2 | 29.15 | 274.203 | .888 | .993 |
| DASS-21 Q3 | 29.21 | 273.892 | .930 | .993 |
| DASS-21 Q4 | 29.25 | 274.209 | .935 | .993 |
| DASS-21 Q5 | 29.25 | 275.134 | .944 | .993 |
| DASS-21 Q6 | 29.27 | 274.772 | .949 | .993 |
| DASS-21 Q7 | 29.30 | 275.517 | .940 | .993 |
| DASS-21 Q8 | 29.15 | 273.830 | .884 | .993 |
| DASS-21 Q9 | 29.22 | 274.065 | .940 | .993 |
| DASS-21 Q10 | 29.27 | 274.587 | .950 | .993 |
| DASS-21 Q11 | 29.26 | 273.963 | .960 | .993 |
| DASS-21 Q12 | 29.27 | 274.012 | .958 | .993 |
| DASS-21 Q13 | 29.27 | 274.570 | .953 | .993 |
| DASS-21 Q14 | 29.24 | 275.061 | .925 | .993 |
| DASS-21 Q15 | 29.29 | 274.339 | .963 | .993 |
| DASS-21 Q16 | 29.28 | 274.274 | .958 | .993 |
| DASS-21 Q17 | 29.36 | 276.638 | .910 | .993 |
| DASS-21 Q18 | 29.25 | 276.093 | .886 | .993 |
| DASS-21 Q19 | 29.28 | 274.478 | .933 | .993 |
| DASS-21 Q20 | 29.31 | 275.315 | .930 | .993 |
| DASS-21 Q21 | 29.34 | 275.689 | .920 | .993 |
